# Dose-Response Relationship Between Serum 2,3,7,8-Tetrachlorodibenzo-p-Dioxin and Diabetes Mellitus: A Meta-Analysis

**DOI:** 10.1093/aje/kwu307

**Published:** 2015-03-01

**Authors:** Michael Goodman, K. M. Venkat Narayan, Dana Flanders, Ellen T. Chang, Hans-Olov Adami, Paolo Boffetta, Jack S. Mandel

**Keywords:** Agent Orange, diabetes mellitus, dioxin, dose-response, heterogeneity, meta-analysis, TCDD

## Abstract

We systematically evaluated studies published through May 2014 in which investigators assessed the dose-response relationship between serum levels of 2,3,7,8-tetrachlorodibenzo-p-dioxin (TCDD) and the occurrence of diabetes mellitus (DM), and we investigated the extent and sources of interstudy heterogeneity. The dose-response relationship between serum TCDD and DM across studies was examined using 2 dependent variables: an exposure level–specific proportion of persons with DM and a corresponding natural log-transformed ratio measure of the association between TCDD and DM. Regression slopes for each dependent variable were obtained for each study and included in a random-effects meta-analysis. Sensitivity analyses were used to assess the influence of inclusion and exclusion decisions, and sources of heterogeneity were explored using meta-regression models and a series of subanalyses. None of the summary estimates in the main models or in the sensitivity analyses indicated a statistically significant association. We found a pronounced dichotomy: a positive dose-response in cross-sectional studies of populations with low-level TCDD exposures (serum concentrations <10 pg/g lipid) and heterogeneous, but on balance null, results for prospective studies of persons with high prediagnosis TCDD body burdens. Considering the discrepancy of results for low current versus high past TCDD levels, the available data do not indicate that increasing TCDD exposure is associated with an increased risk of DM.

Although the term “dioxin” is used to represent a variety of related compounds (1), it often refers specifically to 2,3,7,8-tetrachlorodibenzo-p-dioxin (TCDD). Low-level exposures to TCDD are common (2, 3); however, the most informative human data on the potential health effects of this chemical come from studies of persons who encountered unusually high TCDD levels (4). In particular, researchers have extensively studied the US veterans of the Vietnam War who participated in Operation Ranch Hand, which involved aerial spraying of Agent Orange, a mixture of herbicides contaminated with TCDD (5–10). These veterans formed the core of the Air Force Health Study (AFHS), which began in 1982 and involved extensive periodic physical examinations of the Operation Ranch Hand cohort and the comparison subcohorts over a 20-year follow-up period (11).

Among the diseases reported to be associated with TCDD exposure in the AFHS cohort is diabetes mellitus (DM) (12–15). After publication of the AFHS results, the possible association between TCDD and DM drew considerable attention. In 1999, in response to a request from the Department of Veterans Affairs, the Institute of Medicine convened a committee to review the evidence regarding the possible association of TCDD and other chemical compounds found in the herbicides used in Vietnam with DM. Although the conclusions of the Institute of Medicine were not restricted specifically to TCDD, the committee characterized the available evidence as “limited/suggestive” (16). This designation remained in place when the Institute of Medicine re-examined the data in 2012 (17).

The association between DM and dioxins was examined in several reviews (18–21) that considered, but did not specifically focus on, TCDD data. None of the previous reviews contained a quantitative assessment of the dose-response relationship between TCDD body burdens and DM across the available studies. Such analyses might be important because levels of measured TCDD range widely among exposed populations (22).

In view of the existing knowledge gaps, we conducted a meta-analysis with 2 main objectives. The first objective was to systematically evaluate the evidence regarding the dose-response relationship between blood levels of TCDD and DM occurrence (i.e., prevalence and/or incidence). The second objective was to examine agreement across studies and to identify study characteristics or specific studies that might act as sources of heterogeneity.

## METHODS

### Literature search

We conducted the initial literature search using the PubMed, Ovid, EMBASE, and Google Scholar electronic databases using the following combinations of general text keywords and Medical Subject Headings (MeSH) terms: “tetrachlorodibenzodioxin”[MeSH Terms] OR “tetrachlorodibenzodioxin”[All Fields] OR “dioxin”[All Fields] OR “dioxins”[MeSH Terms] OR “dioxins”[All Fields] OR “tcdd”[All Fields]) AND (“diabetes mellitus”[MeSH Terms]) OR (“diabetes”[All Fields] AND “mellitus”[All Fields]) OR “diabetes mellitus”[All Fields] OR “diabetes”[All Fields]. Secondary references of retrieved articles and recent reviews were examined to identify publications not captured in the electronic search. Additional searches were conducted to identify relevant reports that were not published in the peer-reviewed literature. A list of studies retrieved and evaluated but excluded from the review, as well as the reasons for exclusions, are provided in the Web Appendix (available at http://aje.oxfordjournals.org/). Two study authors (K.M.V.N. and M.G.) conducted the search of relevant studies, with all disagreements resolved by consensus.

### Selection of studies and data extraction

The criteria for inclusion into the present review were as follows.

- TCDD exposure was measured in blood samples and expressed as picograms per gram of lipid;
- Proportions of subjects with prevalent or incident DM were reported for each exposure category; and
- TCDD levels in each exposure category were categorized based on reported cutoffs or (preferably) on a measure of central tendency (mean, median, or geometric mean).

For populations with high levels of exposure, we used TCDD levels measured in archived samples or levels that were back-extrapolated (by the original study authors) from current TCDD concentrations. For populations with background or low-level exposure (serum concentrations of <10 pg/g lipid), only current TCDD concentrations were available.

In some studies, the authors did not document the measures of central tendency but instead reported category cutoffs. When extracting information from those studies, we assigned midpoints between cutoff points to each category except the highest. For the highest exposure category, we used the lower bound for that category plus the width of the preceding interval. This approach was previously shown to adequately approximate the slope compared with individual-level analyses for other exposures and was recommended in meta-analyses when the category-specific measures of central tendency are not available (23). Median levels for each exposure category in 1 study (24) were obtained from the authors via a personal communication.

In addition to extracting information on serum TCDD levels, we retrieved data on the type of study population and the circumstances of exposure. We also characterized each study with respect to its design, sample size, type and case definition of DM, and consideration of confounders in the analysis.

### Dose-response analyses

We examined the dose-response relationship of serum levels of TCDD with DM across studies using models with 2 alternative dependent variables: 1) a proportion (prevalence or incidence) of persons with DM in each exposure category, denoted *P*(DM); or 2) an exposure level–specific natural log-transformed ratio measure of association (i.e., relative risk) between TCDD and DM, denoted ln(RR). The estimates for *P*(DM) were calculated on the basis of the information abstracted from eligible studies; for 1 study (25), they were obtained from the authors through a personal communication. The standard errors for each of these proportion estimates were calculated as

$$\sqrt{\frac{P(\text{DM})\times q}{N}},$$

where *q* = 1 − *P*(DM) and *N* = total number of subjects.

Ratio-based measures of association (prevalence ratios, risk or rate ratios, or odds ratios) and the corresponding 95% confidence intervals were also abstracted from the original articles. For simplicity, all ratio-based measures of association in this meta-analysis are referred to as relative risk. Whenever available, adjusted relative risk estimates were used. If these measures were not reported in an article, the crude relative risk estimates and 95% confidence intervals were calculated by one of the authors (M.G.) using OpenEpi statistical software (26).

The meta-analyses for each of the above dependent variables were conducted using several approaches. First, meta-regression slopes were used to describe the overall relationship between serum levels of TCDD (picogram per gram of lipid) across all studies and each of the outcome measures—*P*(DM) or ln(RR). The independent variable in the analyses was the difference between the highest exposure category and the reference category, and the dependent variable was either the difference between category-specific *P*(DM) estimates or the corresponding ln(RR). The results of these meta-regression analyses were presented graphically and expressed numerically as regression coefficients and the corresponding 95% confidence interval.

Second, to take advantage of all available data while avoiding the problem of within-study correlated observations, we obtained separate regression slopes for each study (using 2 alternative dependent variables, *P*(DM) and ln(RR), as described above) and then included study-specific β coefficients and variance estimates in a meta-analysis. When available, the study-specific regression coefficients were obtained directly from the articles. Otherwise, the dose-response coefficients for *P*(DM) for each study were calculated by constructing a variance-weighted least-squares slope, which is the equivalent of a fixed-effects meta-regression (27). The corresponding study-specific coefficients for ln(RR) were calculated using methods proposed and applied elsewhere previously (28, 29). Individual study-specific slope estimates were combined into a summary meta-analysis using random-effects models with results expressed as summary regression coefficients and 95% confidence intervals.

Whenever 2 or more studies were conducted using the same data, the default approach was to include results based on TCDD levels before the DM diagnosis (e.g., back-extrapolated by the original study authors or measured using archived samples) and to incorporate adjusted measures of association as reported in the original study (rather than values calculated from the data provided in the paper). To assess the influence of these decisions on the observed summary estimates, we conducted sensitivity analyses by comparing the results obtained with alternative input parameters to those from the default model. Because some of the studies used external reference groups and some conducted within-study analyses, the default approach was to include within-study estimates for consistency. As part of sensitivity analyses, however, we obtained the results with inclusion of external comparisons (if available).

In addition to treating serum TCDD as a categorical or a continuous variable, some studies also reported measures of association for TCDD concentrations using log_10_, log_2_, or natural log transformations. As part of sensitivity analyses, we converted log_2_- and log_10_-transformed values to natural logarithm-based measures and calculated the meta-ln(RR) estimate per natural-log change in serum TCDD.

### Assessment of heterogeneity and evaluation of publication bias

All meta-analysis models were accompanied by tests for heterogeneity (30). We also calculated the *I*^2^ statistic, which gives the percentage of the total variation across studies due to heterogeneity, with values of 25%, 50%, and 75% considered as cutoffs for low, moderate, and high levels of heterogeneity, respectively (31).

To explore sources of heterogeneity, we constructed additional meta-regression models in which the study-specific regression coefficients served as dependent variables; independent variables were various study characteristics, including exposure circumstances (military, industrial, or background nonoccupational), measure of DM occurrence (incidence vs. prevalence), crude versus adjusted measures of association (for ln(RR) analyses only), use of prediagnosis (measured or back-extrapolated) serum TCDD concentrations versus current levels to characterize exposure, and DM definition. In the presence of a statistically significant association between study characteristics and observed results, additional subanalyses were carried out to further explore the sources of heterogeneity.

Publication bias was examined by inspecting funnel plots (32) and by performing the Egger's test for the effect of small size studies (33). All analyses were performed using Stata statistical software (version 13.1; StataCorp LP, College Station, Texas) and Episheet, an Excel-based statistical calculator that is available at http://www.krothman.org/Episheet.xls.

## RESULTS

### Overview of the available data

In the present meta-analysis, we incorporated information from 10 epidemiologic studies in which the associations between serum TCDD levels and DM occurrence were assessed (Web Table 1). The articles that met eligibility criteria described 8 different populations with some data overlap. Of the 10 studies, 3 examined workers occupationally exposed to high levels of TCDD (34–36), 3 provided data on the Operation Ranch Hand cohort (12, 34, 37), 3 evaluated Vietnam War veterans with low-level exposures who had not participated in Operation Ranch Hand (38–40), 1 assessed women exposed to TCDD in a residential setting after an industrial accident in Seveso, Italy (24), and 1 was based on a sample of persons in the general Japanese population (25). In 1 of the 10 studies (34), data from 2 different populations were analyzed—Operation Ranch Hand veterans and manufacturers of 2,4,5-trichlorophenol and Agent Orange who comprised an occupational cohort evaluated by the US National Institute for Occupational Safety and Health (NIOSH).

With respect to study design, only 1 of the articles in the meta-analysis reported the relationship between measured prediagnosis serum TCDD level and DM incidence rate (24). One other study examined cumulative incidence (risk) of DM in relation to back-extrapolated serum TCDD levels based on measurements taken either before or after DM diagnosis (12), 2 investigated the association between exposure before diagnosis and the prevalence of DM (34, 35), and all others assessed current or postdiagnosis serum TCDD levels and current DM status.

In the study participants, measured serum TCDD concentrations ranged from 0 pg/g to 17,300 pg/g of lipid, with the highest concentration detected among women in the Seveso Women's Health Study (41). With back-extrapolation of current levels, the highest TCDD concentration was 19,744 pg/g, as reported in the NIOSH cohort (34). In studies that included background levels, the difference between the minimum and the maximum serum TCDD concentration never exceeded 10 pg/g of lipid.

The definition of DM varied across studies. Of the studies in which DM was ascertained at least in part on the basis of laboratory assessment, the AFHS (12, 37, 40) performed glucose challenge tests, the NIOSH study (34, 36) measured fasting blood glucose concentration, and the Seveso Women's Health Study (24) used both fasting blood glucose and glycosylated hemoglobin levels. Of the studies that did not perform any laboratory analyses, 1 (35) relied exclusively on diagnostic codes and 2 (25, 38) used self-reports; in another study, (39) participants underwent “standardized comprehensive clinical investigation,” but the case definition for DM was not provided. Only AFHS investigators specifically indicated that all of the cases in their study had type 2 DM. Otherwise, no studies separately analyzed type 1 and type 2 DM.

### Dose-response analyses for the highest versus lowest categories of TCDD exposure

**Figure 1.**
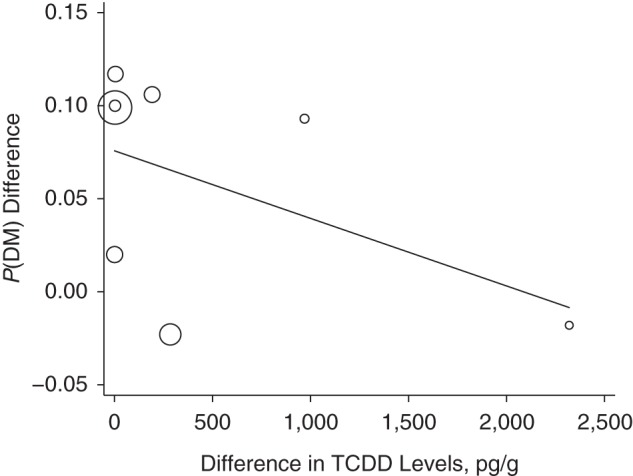
Results of a meta-regression for the difference in the proportion of persons with diabetes (*P*(DM)) between the highest and lowest exposure categories (*y*-axis) by the corresponding difference in levels of 2,3,7,8-tetrachlorodibenzo-p-dioxin (TCDD) (*x*-axis). In the main analysis, in which we used data from Steenland et al. (34) (National Institute of Occupational Safety and Health cohort), Zober et al. (35), Henriksen et al. (12), Warner et al. (24), Kim et al. (39), Longnecker and Michalek (40), Kang et al. (38), and Nakamoto et al. (25), β =−0.00004 (95% confidence interval (CI): −0.00012, 0.00004; *P* =0.310). In the sensitivity analysis in which data from Henriksen et al. (12) were replaced with data from the US Air Force (37), β = −0.00003 (95% CI: −0.00011, 0.00005; *P* = 0.339). In the sensitivity analysis in which within-study analyses were replaced with results using an external reference group (if available), β = −0.00003 (95% CI: −0.00009, 0.00003; *P* = 0.285). This analysis was limited to 7 observations because participants in the study by Longnecker and Michalek (40) served as an external comparison group for the Operation Ranch Hand cohort.

**Figure 2.**
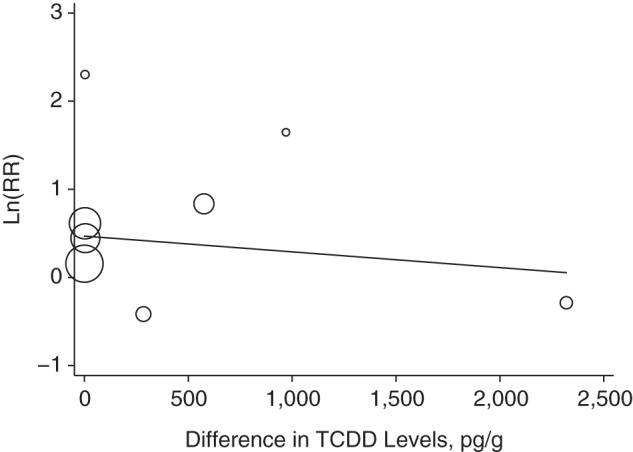
Results of a meta-regression for natural log-transformed relative risk estimate (ln(RR)) (*y*-axis) by maximum difference between the highest and the lowest levels of 2,3,7,8-tetrachlorodibenzo-p-dioxin (TCDD) (*x*-axis). In the main analysis, in which we used data from Steenland et al. (34) (National Institute of Occupational Safety and Health cohort), Zober et al. (35), Steenland et al. (34) (Operation Ranch Hand cohort), Warner et al. (24), Kim et al. (39), Longnecker and Michalek (40), Kang et al. (38), and Nakamoto et al. (25), β = −0.00018 (95% confidence interval (CI): −0.00107, 0.00072; *P* = 0.643). In the sensitivity analysis in which Steenland et al. (34) Operation Ranch Hand cohort data were replaced with data from Henriksen et al. (12), β = −0.00026 (95% CI: −0.00117, 0.00065; *P* = 0.515). In the sensitivity analysis in which Steenland et al. (34) Operation Ranch Hand cohort data were replaced with data from the US Air Force (37), β = −0.00027 (95% CI: −0.00115, 0.00060; *P* = 0.476). In the sensitivity analysis in which within-study analyses were replaced with results using an external reference group (if available), β = −0.00020 (95% CI: −0.00084, 0.00044; *P* = 0.456). This analysis was limited to 7 observations because participants from the study by Longnecker and Michalek (40) served as an external comparison group for the Operation Ranch Hand cohort.

The relationship of the maximum difference between TCDD concentrations in the highest versus the lowest exposure category with the corresponding difference in *P*(DM) is shown in Figure 1. The resulting regression slope was not statistically significantly different from the null value (β = −0.00004; 95% confidence interval (CI): −0.00012, 0.00004; *P* = 0.31). In the corresponding analyses with ln(RR) as the outcome (Figure 2), the summary regression coefficient was also not statistically significant (β = −0.00018; 95% CI: −0.00107, 0.00072; *P* = 0.64). The results of sensitivity analyses did not materially affect the direction or the precision of the original estimates (Figures 1 and 2).

### Meta-analysis of study-specific regression coefficients

**Figure 3.**
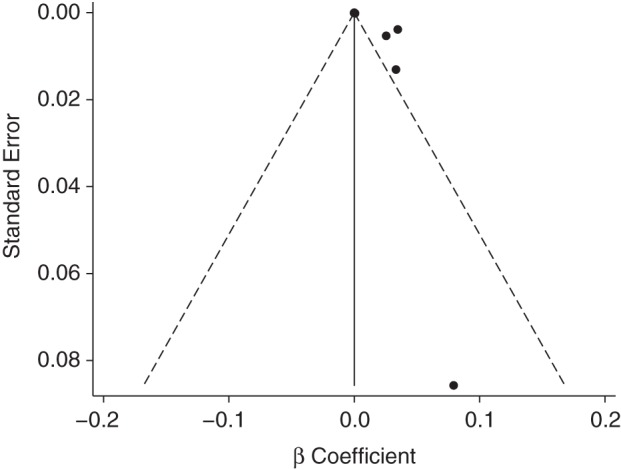
Funnel plot of study-specific regression coefficients using the proportion with diabetes mellitus as the dependent variable. Egger test *P* = 0.026. The studies that were included were Steenland et al. (34) (National Institute of Occupational Safety and Health cohort), Zober et al. (35), Warner et al. (24), Henriksen et al. (12), Longnecker and Michalek (40), Kang et al. (38), Kim et al. (39), and Nakamoto et al. (25).

As shown in Table 1, when we combined individual measures of association in a random-effects meta-analysis, the summary estimate was 0.00021 (95% CI: −0.00012; 0.00053; *P* = 0.21). This result is difficult to interpret because of marked interstudy heterogeneity, with a *Q* statistic *P* value of <0.001 and an *I*^2^ of 94.9%. In the presence of significant heterogeneity, the interpretation of the Egger test (*P* = 0.026) is also unclear, although the corresponding funnel plot appeared asymmetric (Figure 3).

**Table 1.** Study-Specific Regression Coefficients From a Meta-Analysis Using the Proportion of Persons With Diabetes Mellitus as the Dependent Variable

First Author, Year (Reference)	Study Cohort	β	95% CI
Steenland et al., 2001 (34)	NIOSH cohort	−0.00001	−0.00005, 0.00003
Zober et al., 1994 (35)	BASF cohort	0.00010	0.00000, 0.00019
Warner et al., 2013 (24)	Seveso Women's Health Study cohort	−0.00012	−0.00022, −0.00002
Henriksen et al., 1997 (12)	Operation Ranch Hand cohort	0.00055	0.00027, 0.00083
Longnecker and Michalek, 2000 (40)	Air Force veterans not in Operation Ranch Hand	0.02544	0.01502, 0.03586
Kang et al., 2006 (38)	US Army Chemical Corps	0.03333	0.00772, 0.05895
Kim et al., 2003 (39)	Korean Vietnam War veterans	0.07922	−0.08880, 0.24724
Nakamoto et al., 2013 (25)	Japanese general population	0.03474	0.02723, 0.04225
Summary estimate^a^		0.00021	−0.00012, 0.00053

Abbreviations: BASF, Badische Anilin- und Soda-Fabrik; CI, confidence interval; NIOSH, National Institute of Occupational Safety and Health.

^a^ Tests for heterogeneity: *Q* statistic, *P* < 0.001; *I*^2^ = 94.9%.

**Figure 4.**
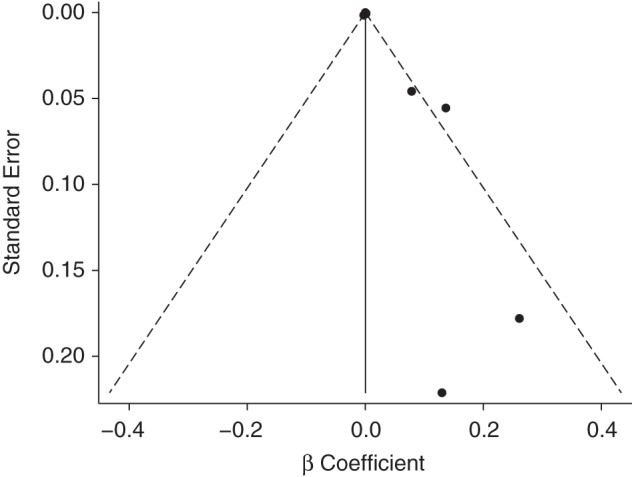
Funnel plot of study-specific regression coefficients using the natural log-transformed relative risk estimate as the dependent variable. Egger test *P* = 0.089. The studies that were included were Steenland et al. (34) (National Institute of Occupational Safety and Health cohort), Zober et al. (35), Warner et al. (24), Steenland et al. (34) (Operation Ranch Hand cohort); Longnecker and Michalek (40), Kang et al. (38), Kim et al. (39), and Nakamoto et al. (25).

Table 2 presents the results of the meta-analysis in which ln(RR) was the dependent variable. In general, the findings were consistent with those for *P*(DM). The overall summary estimate was not statistically significant (β = 0.00055; 95% CI: −0.00053, 0.00163; *P* = 0.39) but represented a weighted average of significantly heterogeneous results (*P* for heterogeneity = 0.001; *I*^2^ = 72.0%) The Egger test *P* value was 0.089, and the funnel plot (Figure 4) did not appear symmetric on visual inspection.

**Table 2.** Study-Specific Regression Coefficients From a Meta-Analysis Using the Natural Log-Transformed Relative Risk Estimate as the Dependent Variable

First Author, Year (Reference)	Study Cohort	β	95% CI
Steenland et al., 2001 (34)	NIOSH cohort	0.00020	0.00005, 0.00035
Zober et al., 1994 (35)	BASF cohort	0.00135	−0.00011, 0.00282
Warner et al., 2013 (24)	Seveso Women's Health Study cohort	−0.00258	−0.00555, 0.00040
Steenland et al., 2001 (34)	Operation Ranch Hand	0.00130	0.00055, 0.00205
Longnecker and Michalek, 2000 (40)	Air Force veterans not in Operation Ranch Hand	0.07870	−0.01115, 0.16856
Kang et al., 2006 (38)	US Army Chemical Corps	0.13667	0.02778, 0.24556
Kim et al., 2003 (39)	Korean or Vietnam War veterans	0.13016	−0.30336, 0.56369
Nakamoto et al., 2013 (25)	Japanese general population	0.26101	−0.08788, 0.60990
Summary estimate^a^		0.00055	−0.00053, 0.00163

Abbreviations: BASF, Badische Anilin- und Soda-Fabrik; CI, confidence interval; NIOSH, National Institute of Occupational Safety and Health.

^a^ Tests for heterogeneity: *Q* statistic, *P* = 0.001; *I*^2^ = 72.0%.

The results of sensitivity analyses in which we compared the default model to various alternative approaches are presented in Table 3. When the regression coefficients for back-extrapolated serum TCDD level in the Operation Ranch Hand and NIOSH cohorts were replaced with the corresponding results for the current TCDD levels, the summary estimate became noticeably greater in magnitude (0.00142 vs. 0.00055), although it remained statistically nonsignificant.

**Table 3.** Sensitivity Analyses Assessing the Influence of Different Inclusion Decisions on the Summary Estimates and Measures of Heterogeneity

Model	Summary Estimate		Tests for Heterogeneity	
	β	95% CI	*P*	*I*^2^
Models for *P*(DM)				
Original model	0.00021	−0.00012, 0.00053	<0.001	94.9%
Replacing Henriksen et al. (12) with USAF (37)	0.00010	−0.00021, 0.00041	<0.001	94.3%
Using results for external comparisons (if available)^a^	0.00012	−0.00015, 0.00039	<0.001	95.1%
Models for ln(RR)				
Original model	0.00055	−0.00053, 0.00163	0.001	72.0%
Replacing Steenland et al. (34) with Henriksen et al. (12)	0.00077	−0.00093, 0.00247	<0.001	73.6%
Replacing Steenland et al. (34) with USAF (37)	0.00027	−0.00081, 0.00136	0.016	59.5%
Using current TCDD levels for the NIOSH and the Operation Ranch Hand cohorts from Steenland et al. (34)	0.00142	−0.00082, 0.00365	<0.001	74.8%
Using results for external comparisons (if available)^a^	0.00029	−0.00095, 0.00153	<0.001	79.7%
Using results for ln-transformed serum TCDD levels^b^	0.06947	−0.24207, 0.38101	0.029	71.8%

Abbreviations: CI, confidence interval; ln(RR), natural log-transformed relative risk estimate; NIOSH, National Institute of Occupational Safety and Health; *P*(DM), proportion with diabetes mellitus; TCDD, 2,3,7,8-tetrachlorodibenzo-p-dioxin; USAF, United States Air Force.

^a^ This analysis was limited to 7 observations because participants from the study by Longnecker and Michalek (40) served as an external comparison group for the Operation Ranch Hand cohort.

^b^ This analysis was based on 3 studies that reported results for log-transformed exposure levels: Steenland et al. (34) for the NIOSH cohort, USAF (37) for the Operation Ranch Hand cohort, and Warner et al. (24) for the Seveso Women's Health Study. Log_10_ and log_2_ values were converted to ln-based measures for consistency.

### Examination of reasons for heterogeneity

**Figure 5.**
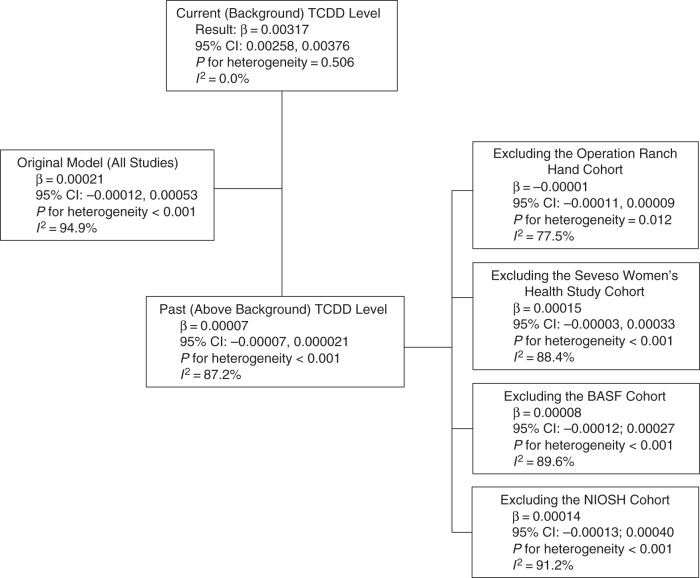
Exploration of interstudy heterogeneity for meta-analysis models that used proportion with diabetes mellitus as the outcome of interest. The studies that were included were Steenland et al. (34) (National Institute of Occupational Safety and Health (NIOSH) cohort), Zober et al. (35), Warner et al. (24), Henriksen et al. (12), Longnecker and Michalek (40), Kang et al. (38), Kim et al. (39), and Nakamoto et al. (25). BASF, Badische Anilin- und Soda-Fabrik; CI, confidence interval; TCDD, 2,3,7,8-tetrachlorodibenzo-p-dioxin.

**Figure 6.**
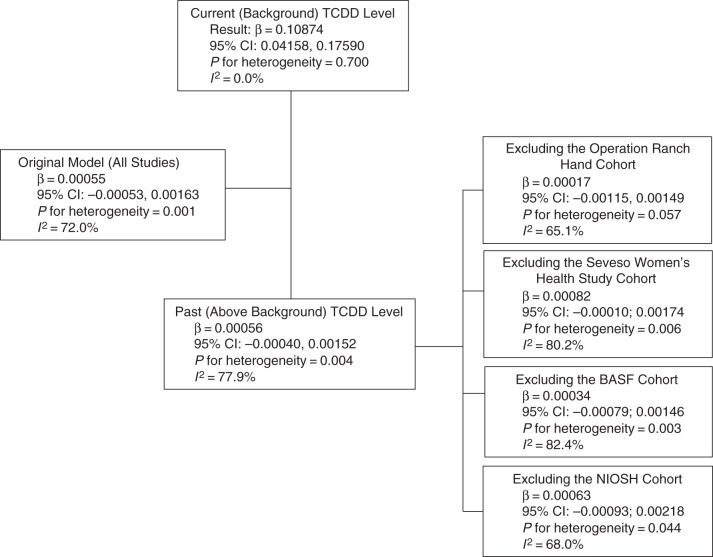
Exploration of interstudy heterogeneity for meta-analysis models that used natural log-transformed relative risk estimates as the outcome of interest. The studies that were included were Steenland et al. (34) (National Institute of Occupational Safety and Health (NIOSH) cohort), Zober et al. (35), Warner et al. (24), Steenland et al. (34) (Operation Ranch Hand cohort); Longnecker and Michalek (40), Kang et al. (38), Kim et al. (39), and Nakamoto et al. (25). BASF, Badische Anilin- und Soda-Fabrik; CI, confidence interval; TCDD, 2,3,7,8-tetrachlorodibenzo-p-dioxin.

In the meta-regression analyses in which we assessed the relationship of the study-specific dose-response slopes with methodological and population-related characteristics (Table 4), the most notable result was the statistically significant and inverse association between the type of exposure assessment (past high level vs. current low level) and the reported dose-response slope. We explored this association further by conducting a series of subanalyses (Figures 5 and 6).

**Table 4.** Results From Meta-Regression Analyses Assessing the Association Between Methodological Characteristics and Observed Results in Each Study

Study Characteristic	Models for *P*(DM)		Models for ln(RR)	
	Meta-Regression Coefficient	95% CI	Meta-Regression Coefficient	95% CI
Incidence vs. prevalence estimates for DM	−0.02148	−0.04540, 0.00244	−0.00001	−0.00717, 0.00714
Veterans vs. other study populations	0.01004	−0.02258, 0.04266	0.00135	−0.00435, 0.00705
Occupational groups vs. other study populations	−0.01730	−0.04817, 0.01357	0.00062	−0.00574, 0.00697
Industrial vs. other exposures	−0.02178	−0.04531, 0.00174	−0.00137	−0.00711, 0.00436
Laboratory test–based DM assessment vs. other methods	−0.01542	−0.04544, 0.01461	−0.00130	−0.00764, 0.00503
Adjusted vs. unadjusted results	N/A	N/A	−0.00112	−0.00246, 0.00022
Past (above background) vs. current (background) TCDD levels	−0.03161	−0.03903, −0.02420	−0.10818	−0.19350, −0.02276

Abbreviations: CI, confidence interval; DM, diabetes mellitus; ln(RR), natural log-transformed relative risk estimate; *P*(DM), proportion with diabetes mellitus; TCDD, 2,3,7,8-tetrachlorodibenzo-p-dioxin.

When the meta-analysis was limited to studies of populations with current low-level exposures, the summary estimate for *P*(DM) became stronger (β = 0.00317), statistically significant (95% CI: 0.00258, 0.00376), and based on highly homogeneous results (*I*^2^ = 0%). In contrast, for high-level past exposure studies, the summary estimate was essentially 0 (β = 0.00007; 95% CI: −0.00007, 0.000021), with only minimal reduction in heterogeneity (*I*^2^ = 87.2%). The corresponding subanalyses of the ln(RR) data were similar (Figure 6).

## DISCUSSION

Meta-analysis was originally proposed and is still often used as a way of integrating findings from multiple studies to produce an overall numeric result (42, 43). Although meta-analytic techniques allow calculation of a summary estimate for a particular measure of effect, the interpretation of this summary estimate might be difficult and sometimes impossible because of disagreement across results, differences in study methods, evidence of selective reporting (also known as publication bias), or low quality of the available data (32, 44). Nevertheless, even when a summary measure of association does not allow a simple overall conclusion, meta-analysis might still provide important information about patterns of results and their relation to study characteristics (45–47).

With these considerations in mind, the main contribution of the present study is that it offers a systematic quantitative assessment of the extent and sources of disagreement across studies. Perhaps the most noteworthy finding of this meta-analysis is the pronounced difference between a homogeneous and statistically significant positive dose-response among populations with common low-level TCDD exposures and rather heterogeneous but weak and statistically nonsignificant results for studies of persons with high TCDD body burdens. The observed dichotomy might have several explanations because the 2 categories of studies differed in a number of ways besides the levels of exposure.

A critical—and perhaps the only inarguable—property of analytic epidemiologic studies aimed at assessing causal associations is the ability to establish the proper temporal sequence of exposure and outcome, either through follow-up or through reconstruction of exposures preceding the outcome of interest (48, 49). It is important to note that of the studies included in the present review, only those in the high-TCDD category included analyses of past (prediagnosis) levels.

In the absence of an established exposure-disease sequence, the direction of the association between TCDD and DM is almost impossible to ascertain. Overt DM produces intermittent lipolysis, which in turn may release tissue dioxin into the bloodstream, thereby leading to elevated TCDD levels (50). The possibility of reverse causation in the association between TCDD and DM has been discussed specifically in relation to data from the Operation Ranch Hand cohort (50, 51). However, reverse causation should be of even greater concern in cross-sectional studies with relatively narrow ranges of exposure. Even in the absence of reverse causation, body fat, which stores TCDD, might act as a confounder or effect modifier of the association between TCDD and DM. Most studies controlled for body mass index (BMI); however, the BMI values used in those studies did not necessarily reflect the BMI before the onset of DM. Moreover, BMI is not a good measure of fat deposits. In studies of whole-body magnetic resonance imaging, it has been demonstrated that persons with similar BMIs might have very different volumes of visceral fat (52). Visceral fat is a stronger predictor of metabolic problems, including diabetes, than is somatic fat or BMI (53), and BMI as a measure of body fat deposits has been shown to be particularly inadequate in men (54). Consequently, controlling for BMI (even if measured at baseline) might not eliminate confounding by body fat deposits, which might result in bias away from the null. Future investigations might profit from a pooled analysis of individual level data to further explore the confounding or effect-modifying influence of adiposity on any potential association between TCDD and DM.

As all dioxins are highly fat-soluble (55), it follows that the association between TCDD levels and DM might be affected by confounding bias from different amounts of circulating blood lipids. Mindful of this issue, the authors of all studies included in the present meta-analysis expressed TCDD concentrations per gram of blood lipids; however, this approach might still leave room for error. As discussed in the study by Longnecker and Michalek (40), dioxin is more soluble in certain blood lipids (e.g., triglycerides) than in others, and for this reason it could be important to account for the blood lipid composition rather than total serum fat. Controlling for triglyceride level in addition to other covariates appreciably attenuated, although did not explain away, the TCDD-DM association in that study (40). Other studies did not control for triglycerides or other blood lipids.

Although analyses using back-extrapolated TCDD concentrations are preferable to those using current levels, they remain subject to uncertainty and might underestimate the baseline levels by several fold (1, 56). Moreover, the concern about reverse causation also applies to back-extrapolated TCDD levels based on serum samples collected shortly before or after DM diagnosis, because serum TCDD concentrations might be affected by DM status. Thus, the only way to obtain accurate estimates of prediagnosis TCDD body burdens and avoid the problem of reverse causation is to measure TCDD in serum samples obtained before the onset of DM.

A formal quantitative examination of the impact of measured versus back-extrapolated TCDD exposure was not possible because of the 10 publications included in the present meta-analysis, only the Seveso Women's Health Study (24) relied on measured rather than estimated past exposures. The association between serum TCDD and DM in that study was inverse but not statistically significant.

Among prospective studies, the main disagreement was observed between the positive AFHS results and the null findings in the similarly well-conducted NIOSH and Seveso Women's Health studies. This disagreement is not attributable to the differences in the levels of exposure because Operation Ranch Hand veterans had TCDD concentrations that were orders of magnitude lower than those in the other 2 cohorts. It is possible that AFHS exposures were qualitatively different; yet, TCDD was the only chlorinated chemical significantly elevated in blood samples of Operation Ranch Hand veterans (57), and the NIOSH study participants were also exposed to Agent Orange (34). Another difference between the AFHS and other studies is the case definition of DM, which was based on a glucose tolerance test only in the AHFS, but it is unlikely that this methodological feature explains the heterogeneity of results. It is more likely that the disagreement is explained by the differences in the study populations, yet-unidentified differences in study methods, or chance.

Although the relatively small number of observations and the pronounced interstudy heterogeneity precluded a formal evaluation of publication bias, the funnel plots appeared asymmetrical. The most pronounced positive effect sizes appeared to be reported in studies with the lowest precision of estimates.

In preparation for the present meta-analysis, we reviewed a number of studies that did not meet the inclusion criteria. These studies are summarized in the Web Appendix. The reasons for exclusion fell into 3 main categories: 1) lack of TCDD exposure assessment or no data on serum TCDD levels; 2) use of laboratory test results but not DM as the endpoint of interest; and 3) evaluation of mortality from DM rather than DM incidence or prevalence.

Although cause-specific mortality is an inadequate measure of DM risk because many factors affect the risk of death (16), it is worth mentioning studies that provided information about the dose-response relationship between TCDD exposure and DM deaths, particularly among cohorts not included in the current review. In a large multicenter study, Vena et al. (58) investigated deaths from causes other than cancer among workers who produced phenoxyacid herbicide and chlorophenol by pooling data from 36 cohorts assembled in 12 countries. Exposure to TCDD or higher levels of chlorinated dioxins was estimated from job records and questionnaires, and duration of exposure was categorized as <1, 1–4, 5–9, 10–19, or ≥20 years. Using less than 1 year of exposure to TCDD/higher chlorinated dioxins as the reference group, the risk ratios for death from DM for each subsequent category were 1.07, 1.01, 2.52, and 1.13, respectively; none of these estimates was statistically significant, and the *P* value for trend was 0.18. Two other relevant cohort mortality studies were conducted at trichlorophenol production facilities in Michigan and New Zealand (59, 60). The data from the New Zealand cohort were previously included in the multicenter study by Vena et al. (58). In both the Michigan and the New Zealand studies, cumulative TCDD exposure was calculated by integrating work history information for each employee with data on serum levels measured in a subset of participants for whom blood samples were available. Neither study found evidence of a significant dose-response relationship between cumulative TCDD exposure and rates of death from DM.

It is important to emphasize that the findings of the present review apply only to the relatively narrow question of the dose-response relationship between serum TCDD levels and DM. These results should not be extrapolated to related chemicals, such as polychlorinated dibenzo-p-dioxins or polychlorinated dibenzofurans. As shown by Nakamoto et al. (25), for populations with background exposures, non-TCDD compounds contribute a substantial proportion of the total dioxin exposure. However, persons with high serum levels of TCDD are also exposed to these compounds (57).

In summary, the current literature on the association between TCDD and measures of DM occurrence is heterogeneous and includes few studies in which the temporality issues could be adequately addressed. The positive dose-response relationship is consistently present in cross-sectional studies with current TCDD levels less than 10 pg/g. In contrast, cohort studies in which populations had measured or estimated TCDD levels that reflected exposures before DM onset and spanned thousands of picograms per gram of lipids provide no clear evidence of a dose-response relationship. Taken together, the available data do not indicate that a higher TCDD exposure is associated with a higher risk of developing DM.

## Supplementary Material

Web Material
