# Supplementary material for: Dose-Response Relationship Between Serum 2,3,7,8-Tetrachlorodibenzo-p-Dioxin and Diabetes Mellitus: A Meta-Analysis
Source: Am J Epidemiol. 2015 Mar 1;181(6):374–84. doi: 10.1093/aje/kwu307 (PMC4380020; doi:10.1093/aje/kwu307)
Supplement: Web Material [file supp_181_6_374__index.html]

Dose-Response Relationship Between Serum 2,3,7,8-Tetrachlorodibenzo-p-Dioxin and Diabetes Mellitus: A Meta-Analysis — Dose-Response Relationship Between Serum 2,3,7,8-Tetrachlorodibenzo-p-Dioxin and Diabetes Mellitus: A Meta-Analysis — Web Material 

# Dose-Response Relationship Between Serum 2,3,7,8-Tetrachlorodibenzo-p-Dioxin and Diabetes Mellitus: A Meta-Analysis

## Web Material

Web Material

**Files in this Data Supplement:**

- Web Material - Pdf file
