# Supplementary material for: Dose-Response Relationship Between Serum 2,3,7,8-Tetrachlorodibenzo-p-Dioxin and Diabetes Mellitus: A Meta-Analysis
Source: Am J Epidemiol. 2015 Mar 1;181(6):374–84. doi: 10.1093/aje/kwu307 (PMC4380020; doi:10.1093/aje/kwu307)
Supplement: Web Material [file supp_kwu307_kwu307supp.pdf]

Web Appendix 1
Articles examined, but excluded from the meta-analysis

Australian Institute of Health and Welfare (AIHW). 1999. Morbidity of Vietnam veterans: A study of the health of Australia’s Vietnam veteran community: Volume 3 Validation study. Canberra: Australian Institute of Health and Welfare.
Reason for exclusion: no TCDD exposure assessment

Airaksinen R, Rantakokko P, Eriksson JG, Blomstedt P, Kajantie E, Kiviranta H. 2011. Association between type 2 diabetes and exposure to persistent organic pollutants. Diabetes Care 34:1972-1979.
Reason for exclusion: no TCDD exposure assessment

Bertazzi PA, Bernucci I, Brambilla G, Consonni D, Pesatori AC. 1998. The Seveso studies on early and long-term effects of dioxin exposure: A review. Environ Health Perspect 106 Suppl 2:625-633.
Reason for exclusion: mortality study

Bertazzi PA, Consonni D, Bachetti S, Rubagotti M, Baccarelli A, Zocchetti C, et al. 2001. Health effects of dioxin exposure: A 20-year mortality study. Am J Epidemiol 153:1031-1044.
Reason for exclusion: mortality study

Boehmer TK, Flanders WD, McGeehin MA, Boyle C, Barrett DH. 2004. Postservice mortality in Vietnam veterans: 30-year follow-up. Arch Intern Med 164:1908-1916.
Reason for exclusion: mortality study; laboratory biomarker study no TCDD exposure assessment

CDC. 1988. Health status of Vietnam veterans. II. Physical health. The Centers for Disease Control Vietnam experience study. JAMA 259:2708-2714.
Reason for exclusion: no TCDD exposure assessment

Chen HL, Su HJ, Guo YL, Liao PC, Hung CF, Lee CC. 2006. Biochemistry examinations and health disorder evaluation of Taiwanese living near incinerators and with low serum PCDD/Fs levels. Sci Total Environ 366:538-548.
Reason for exclusion: laboratory biomarker study

Codru N, Schymura MJ, Negoita S, Akwesasne Task Force on E, Rej R, Carpenter DO. 2007. Diabetes in relation to serum levels of polychlorinated biphenyls and chlorinated pesticides in adult Native Americans. Environ Health Perspect 115:1442-1447.
Reason for exclusion: mortality study; laboratory biomarker study no TCDD exposure assessment

Collins JJ, Bodner K, Aylward LL, Wilken M, Swaen G, Budinsky R, et al. 2009. Mortality rates among workers exposed to dioxins in the manufacture of pentachlorophenol. J Occup Environ Med 51:1212-1219.
Reason for exclusion: mortality study

Consonni D, Pesatori AC, Zocchetti C, Sindaco R, D'Oro LC, Rubagotti M, et al. 2008. Mortality in a population exposed to dioxin after the Seveso, Italy, accident in 1976: 25 years of follow-up. Am J Epidemiol 167:847-858.
Reason for exclusion: mortality study

Cook RR, Bond GG, Olson RA, Ott MG. 1987. Update of the mortality experience of workers exposed to chlorinated dioxins. Chemosphere 16:2111-2116.
Reason for exclusion: mortality study

Cox S, Niskar AS, Narayan KM, Marcus M. 2007. Prevalence of self-reported diabetes and exposure to organochlorine pesticides among Mexican Americans: Hispanic health and nutrition examination survey, 1982-1984. Environ Health Perspect 115:1747-1752.
Reason for exclusion: no TCDD exposure assessment

Crane PJ, Barnard DL, Horsley KD, Adena MA. 1997. Mortality of Vietnam veterans: The veteran cohort study. A report of the 1996 retrospective cohort study of Australian Vietnam veterans. Canberra: Department of Veterans' Affairs.
Reason for exclusion: mortality study

Cranmer M, Louie S, Kennedy RH, Kern PA, Fonseca VA. 2000. Exposure to 2,3,7,8-tetrachlorodibenzo-p-dioxin (TCDD) is associated with hyperinsulinemia and insulin resistance. Toxicol Sci 56:431-436.
Reason for exclusion: laboratory biomarker study

Everett CJ, Frithsen IL, Diaz VA, Koopman RJ, Simpson WM, Jr., Mainous AG, 3rd. 2007. Association of a polychlorinated dibenzo-p-dioxin, a polychlorinated biphenyl, and DDT with diabetes in the 1999-2002 National Health and Nutrition Examination Survey. Environ Res 103:413-418.
Reason for exclusion: no TCDD exposure assessment

Everett CJ, Matheson EM. 2010. Biomarkers of pesticide exposure and diabetes in the 1999-2004 National Health and Nutrition Examination Survey. Environ Int 36:398-401.
Reason for exclusion: no TCDD exposure assessment

Everett CJ, Thompson OM. 2012. Associations of dioxins, furans and dioxin-like PCBs with diabetes and pre-diabetes: Is the toxic equivalency approach useful? Environ Res 118:107-111.
Reason for exclusion: TCDD exposure assessed but association with DM not reported

Fierens S, Mairesse H, Heilier JF, De Burbure C, Focant JF, Eppe G, et al. 2003. Dioxin/polychlorinated biphenyl body burden, diabetes and endometriosis: Findings in a population-based study in Belgium. Biomarkers 8:529-534.
Reason for exclusion: no TCDD exposure assessment

Fujiyoshi PT, Michalek JE, Matsumura F. 2006. Molecular epidemiologic evidence for diabetogenic effects of dioxin exposure in U.S. Air force veterans of the Vietnam War. Environ Health Perspect 114:1677-1683.
Reason for exclusion: laboratory biomarker study

Gasull M, Pumarega J, Tellez-Plaza M, Castell C, Tresserras R, Lee DH, et al. 2012. Blood concentrations of persistent organic pollutants and prediabetes and diabetes in the general population of Catalonia. Environ Sci Technol 46:7799-7810.
Reason for exclusion: no TCDD exposure assessment

Grandjean P, Henriksen JE, Choi AL, Petersen MS, Dalgard C, Nielsen F, et al. 2011. Marine food pollutants as a risk factor for hypoinsulinemia and type 2 diabetes. Epidemiology 22:410-417.
Reason for exclusion: no TCDD exposure assessment

Henneberger PK, Ferris BG, Jr., Monson RR. 1989. Mortality among pulp and paper workers in berlin, New Hampshire. Br J Ind Med 46:658-664.
Reason for exclusion: mortality study

Jorgensen ME, Borch-Johnsen K, Bjerregaard P. 2008. A cross-sectional study of the association between persistent organic pollutants and glucose intolerance among Greenland Inuit. Diabetologia 51:1416-1422.
Reason for exclusion: no TCDD exposure assessment

Karouna-Renier NK, Rao KR, Lanza JJ, Davis DA, Wilson PA. 2007. Serum profiles of PCDDs and PCDFs, in individuals near the Escambia wood treating company superfund site in Pensacola, fl. Chemosphere 69:1312-1319.
Reason for exclusion: no TCDD exposure assessment

Kern PA, Said S, Jackson WG, Jr., Michalek JE. 2004. Insulin sensitivity following agent orange exposure in vietnam veterans with high blood levels of 2,3,7,8-tetrachlorodibenzo-p-dioxin. J Clin Endocrinol Metab 89:4665-4672.
Reason for exclusion: laboratory biomarker study

Ketchum NS, Michalek JE. 2006. A matched analysis of diabetes mellitus and herbicide exposure in veterans of Operation Ranch Hand. Brooks City-Base, TX: United States Air Force.
Reason for exclusion: data overlap with Henriksen et al. (1997), Steenland et al. (2001), Longnecker et al. (2001) and USAF (2005)

Kim KS, Lee YM, Kim SG, Lee IK, Lee HJ, Kim JH, et al. 2014. Associations of organochlorine pesticides and polychlorinated biphenyls in visceral vs. subcutaneous adipose tissue with type 2 diabetes and insulin resistance. Chemosphere 94:151-157.
Reason for exclusion: no TCDD exposure assessment

Kitamura K, Kikuchi Y, Watanabe S, Waechter G, Sakurai H, Takada T. 2000. Health effects of chronic exposure to polychlorinated dibenzo-p-dioxins (PCDD), dibenzofurans (PCDF) and coplanar PCB (co-PCB) of municipal waste incinerator workers. J Epidemiol 10:262-270.
Reason for exclusion: laboratory biomarker study

Langer P, Kocan A, Tajtakova M, Susienkova K, Radikova Z, Koska J, et al. 2009. Multiple adverse thyroid and metabolic health signs in the population from the area heavily polluted by organochlorine cocktail (PCB, DDE, HCB, dioxin). Thyroid Res 2:3.
Reason for exclusion: no TCDD exposure assessment

Lee DH, Lee IK, Jin SH, Steffes M, Jacobs DR, Jr. 2007. Association between serum concentrations of persistent organic pollutants and insulin resistance among nondiabetic adults: Results from the National Health and Nutrition Examination Survey 1999-2002. Diabetes Care 30:622-628.
Reason for exclusion: no TCDD exposure assessment; DM cases excluded

Lee DH, Steffes MW, Sjodin A, Jones RS, Needham LL, Jacobs DR, Jr. 2010. Low dose of some persistent organic pollutants predicts type 2 diabetes: A nested case-control study. Environ Health Perspect 118:1235-1242.
Reason for exclusion: no TCDD exposure assessment

Lee DH, Lind PM, Jacobs DR, Jr., Salihovic S, van Bavel B, Lind L. 2011a. Polychlorinated biphenyls and organochlorine pesticides in plasma predict development of type 2 diabetes in the elderly: The prospective investigation of the vasculature in Uppsala seniors (PIVUS) study. Diabetes Care 34:1778-1784.
Reason for exclusion: no TCDD exposure assessment

Lee DH, Steffes MW, Sjodin A, Jones RS, Needham LL, Jacobs DR, Jr. 2011b. Low dose organochlorine pesticides and polychlorinated biphenyls predict obesity, dyslipidemia, and insulin resistance among people free of diabetes. PLoS One 6:e15977.
Reason for exclusion: no TCDD exposure assessment; DM cases excluded

Luzardo OP, Henriquez-Hernandez LA, Valeron PF, Lara PC, Almeida-Gonzalez M, Losada A, et al. 2012. The relationship between dioxin-like polychlorobiphenyls and IGF-I serum levels in healthy adults: Evidence from a cross-sectional study. PLoS One 7:e38213.
Reason for exclusion: laboratory biomarker study; no TCDD exposure assessment

May G. 1982. Tetrachlorodibenzodioxin: A survey of subjects ten years after exposure. Br J Ind Med 39:128-135.
Reason for exclusion: DM not examined

McBride DI, Collins JJ, Humphry NF, Herbison P, Bodner KM, Aylward LL, et al. 2009. Mortality in workers exposed to 2,3,7,8-tetrachlorodibenzo-p-dioxin at a trichlorophenol plant in New Zealand. J Occup Environ Med 51:1049-1056.
Reason for exclusion: mortality study

Michalek JE, Akhtar FZ, Kiel JL. 1999. Serum dioxin, insulin, fasting glucose, and sex hormone-binding globulin in veterans of Operation Ranch Hand. J Clin Endocrinol Metab 84:1540-1543.
Reason for exclusion: laboratory biomarker study

Michalek JE, Ketchum NS, Tripathi RC. 2003. Diabetes mellitus and 2,3,7,8-tetrachlorodibenzo-p-dioxin elimination in veterans of Operation Ranch Hand. J Toxicol Environ Health A 66:211-221.
Reason for exclusion: analyses focused on TCDD elimination

Michalek JE, Henriksen G, Kulkarni P, Russell IJ, Tripathi RC, Wang S. 2006. The check mark pattern. Brooks City-Base, TX: United States Air Force.
Reason for exclusion: data overlap with Henriksen et al. (1997), Steenland et al. (2001), Longnecker et al. (2001) and USAF (2005)

Michalek JE, Pavuk M. 2008. Diabetes and cancer in veterans of Operation Ranch Hand after adjustment for calendar period, days of spraying, and time spent in Southeast Asia. J Occup Environ Med 50:330-340.
Reason for exclusion: data overlap with Henriksen et al. (1997), Steenland et al. (2001), Longnecker et al. (2001) and USAF (2005)

O'Toole BI, Marshall RP, Grayson DA, Schureck RJ, Dobson M, Ffrench M, et al. 1996. The Australian Vietnam Veterans health study: II. Self-reported health of veterans compared with the Australian population. Int J Epidemiol 25:319-330.
Reason for exclusion: no TCDD exposure assessment

O'Toole BI, Catts SV, Outram S, Pierse KR, Cockburn J. 2009. The physical and mental health of Australian Vietnam Veterans 3 decades after the war and its relation to military service, combat, and post-traumatic stress disorder. Am J Epidemiol 170:318-330.
Reason for exclusion: no TCDD exposure assessment

Ott MG, Zober A, Germann C. 1994. Laboratory results for selected target organs in 138 individuals occupationally exposed to TCDD. Chemosphere 29:2423-2437.
Reason for exclusion: laboratory biomarker study

Ott MG, Zober A. 1996. Morbidity study of extruder personnel with potential exposure to brominated dioxins and furans. II. Results of clinical laboratory studies. Occup Environ Med 53:844-846.
Reason for exclusion: laboratory biomarker study

Pal S, Blais JM, Robidoux MA, Haman F, Krummel E, Seabert TA, et al. 2013. The association of type 2 diabetes and insulin resistance/secretion with persistent organic pollutants in two first nations communities in northern Ontario. Diabetes Metab 39:497-504.
Reason for exclusion: no TCDD exposure assessment

Pazderova-Vejlupkova J, Lukas E, Nemcova M, Pickova J, Jirasek L. 1981. The development and prognosis of chronic intoxication by tetrachlordibenzo-p-dioxin in men. Arch Environ Health 36:5-11.
Reason for exclusion: laboratory biomarker study no TCDD exposure assessment

Pesatori AC, Zocchetti C, Guercilena S, Consonni D, Turrini D, Bertazzi PA. 1998. Dioxin exposure and non-malignant health effects: A mortality study. Occup Environ Med 55:126-131.
Reason for exclusion: mortality study

Pesatori AC, Consonni D, Bachetti S, Zocchetti C, Bonzini M, Baccarelli A, et al. 2003. Short- and long-term morbidity and mortality in the population exposed to dioxin after the "Seveso accident". Ind Health 41:127-138
Reason for exclusion: no TCDD exposure assessment, mortality study

Philibert A, Schwartz H, Mergler D. 2009. An exploratory study of diabetes in a first nation community with respect to serum concentrations of p,p'-DDE and PCB and fish consumption. Int J Environ Res Public Health 6:3179-3189.
Reason for exclusion: laboratory biomarker study no TCDD exposure assessment

Ramlow JM, Spadacene NW, Hoag SR, Stafford BA, Cartmill JB, Lerner PJ. 1996. Mortality in a cohort of pentachlorophenol manufacturing workers, 1940-1989. Am J Ind Med 30:180-194.
Reason for exclusion: mortality study

Rignell-Hydbom A, Rylander L, Hagmar L. 2007. Exposure to persistent organochlorine pollutants and type 2 diabetes mellitus. Hum Exp Toxicol 26:447-452.
Reason for exclusion: no TCDD exposure assessment

Rignell-Hydbom A, Lidfeldt J, Kiviranta H, Rantakokko P, Samsioe G, Agardh CD, et al. 2009. Exposure to p,p'-DDE: A risk factor for type 2 diabetes. PLoS One 4:e7503.
Reason for exclusion: no TCDD exposure assessment

Ruder AM, Yiin JH. 2011. Mortality of US pentachlorophenol production workers through 2005. Chemosphere 83:851-861.
Reason for exclusion: mortality study

Sergeev AV, Carpenter DO. 2011. Increase in metabolic syndrome-related hospitalizations in relation to environmental sources of persistent organic pollutants. Int J Environ Res Public Health 8:762-776.
Reason for exclusion: no TCDD exposure assessment

Son HK, Kim SA, Kang JH, Chang YS, Park SK, Lee SK, et al. 2010. Strong associations between low-dose organochlorine pesticides and type 2 diabetes in Korea. Environ Int 36:410-414.
Reason for exclusion: no TCDD exposure assessment

Steenland K, Piacitelli L, Deddens J, Fingerhut M, Chang LI. 1999. Cancer, heart disease, and diabetes in workers exposed to 2,3,7,8-tetrachlorodibenzo-p-dioxin. J Natl Cancer Inst 91:779-786.
Reason for exclusion: mortality study

Sweeney MH, Hornung RW, Wall DK, Fingerhut MA, Halperin WE. 1992. Diabetes and serum glucose levels in TCDD-exposed workers. In: 12th International Symposium on Chlorinated Dioxins (Dioxin '92). Tampere, Finland.
Reason for exclusion: laboratory biomarker study no TCDD exposure assessment

Sweeney MH, Calvert GM, Egeland GA, Fingerhut MA, Halperin WE, Piacitelli LA. 1997-98. Review and update of the results of the NIOSH medical study of workers exposed to chemicals contaminated with 2,3,7,8-tetrachlorodibenzodioxin. Teratog Carcinog Mutagen 17:241-247.
Reason for exclusion: data overlap with Calvert et al. (1999) and Steenland et al. (2001)

Tanaka T, Morita A, Kato M, Hirai T, Mizoue T, Terauchi Y, et al. 2011. Congener-specific polychlorinated biphenyls and the prevalence of diabetes in the Saku control obesity program (SCOP). Endocr J 58:589-596.
Reason for exclusion: laboratory biomarker study no TCDD exposure assessment

Turyk M, Anderson H, Knobeloch L, Imm P, Persky V. 2009a. Organochlorine exposure and incidence of diabetes in a cohort of Great Lakes sport fish consumers. Environ Health Perspect 117:1076-1082.
Reason for exclusion: no TCDD exposure assessment

Turyk M, Anderson HA, Knobeloch L, Imm P, Persky VW. 2009b. Prevalence of diabetes and body burdens of polychlorinated biphenyls, polybrominated diphenyl ethers, and p,p'-diphenyldichloroethene in Great Lakes sport fish consumers. Chemosphere 75:674-679.
Reason for exclusion: no TCDD exposure assessment

Uemura H, Arisawa K, Hiyoshi M, Satoh H, Sumiyoshi Y, Morinaga K, et al. 2008. Associations of environmental exposure to dioxins with prevalent diabetes among general inhabitants in Japan. Environ Res 108:63-68.
Reason for exclusion: TCDD exposure assessed but association with DM not reported

Ukropec J, Radikova Z, Huckova M, Koska J, Kocan A, Sebokova E, et al. 2010. High prevalence of prediabetes and diabetes in a population exposed to high levels of an organochlorine cocktail. Diabetologia 53:899-906.
Reason for exclusion: no TCDD exposure assessment

Vasiliu O, Cameron L, Gardiner J, Deguire P, Karmaus W. 2006. Polybrominated biphenyls, polychlorinated biphenyls, body weight, and incidence of adult-onset diabetes mellitus. Epidemiology 17:352-359.
Reason for exclusion: no TCDD exposure assessment

Vena J, Boffetta P, Becher H, Benn T, Bueno-de-Mesquita HB, Coggon D, et al. 1998. Exposure to dioxin and nonneoplastic mortality in the expanded IARC international cohort study of phenoxy herbicide and chlorophenol production workers and sprayers. Environ Health Perspect 106 Suppl 2:645-653.
Reason for exclusion: mortality study

Wang SL, Tsai PC, Yang CY, Guo YL. 2008. Increased risk of diabetes and polychlorinated biphenyls and dioxins: A 24-year follow-up study of the Yucheng cohort. Diabetes Care 31:1574-1579.
Reason for exclusion: no TCDD exposure assessment

Wu H, Bertrand KA, Choi AL, Hu FB, Laden F, Grandjean P, et al. 2013. Persistent organic pollutants and type 2 diabetes: A prospective analysis in the Nurses' Health Study and meta-analysis. Environ Health Perspect 121:153-161.
Reason for exclusion: no TCDD exposure assessment

Web Table 1: Summary of studies evaluating the association between TCDD levels and diabetes mellitus

Study; population description and total sample size	Reference(s)	DM measure and definition of diagnosis	TCDD exposure types, categories (N) and category specific measures of central tendency (pg/g lipid)	Proportion with DM (P_{DM})	[illegible]
NIOSH cohort; 267 workers exposed to TCDD at two US TCP manufacturing plants	Steenland et al., 2001 (1) and Calvert et al., 1999 (2)	Prevalence of FBG ≥7.8 mmol/1 on 2 testing days, or self-reported diagnosis	Back-extrapolated levels*<br>10-243 (n=56)† midpoint=126.5<br>243-584 (n=57)† midpoint=413.5<br>584-1,515 (n=57)† midpoint=1049.5<br>1515-19,744 (n=56) † midpoint=2446§<br>* limited to 226 persons with serum TCDD level ≥10 pg/g lipid<br>†estimated based on the data reported in Steenland et al., 2001 (1)<br>§ cutoff + range of the previous interval	 0.107*<br>0.105*<br>0.123*<br>0.089*<br>*estimated based on the data reported in Steenland et al. 2001 (1)	[illegible]
BASF cohort; 158 employees of a German manufacturing facility exposed to TCDD following an industrial accident	Zober et al., 1994 (3)	Prevalence of DM ICD-9 code 250 ever mentioned in medical records	Back-extrapolated levels:<br>No chloracne (n=45); geo. mean=148<br>Moderate chloracne (n=61) geo. mean=421*<br>Severe chloracne (n=52); geo. mean=1118<br>*as reported in Ott et al. 1993 (4)	 0.022<br>0.049<br>0.115	[illegible]
Seveso cohort; 980 women exposed to high TCDD levels in a 1976 chemical plant explosion	Warner et al., 2013 (5)	Incidence of DM defined as self-reported diagnosis after the accident or FBG ≥126 mg/dl or HbA1c ≥6.5%	TCDD measured in samples drawn in 1976<br>≤20 (n=154); median=11.4*<br>20.1-47.0 (n=275); median=31.5*<br>47.1-135.0 (n=278); median=73.1*<br>>135 (n=273); median=296.0*<br>* personal communication with the study authors	 0.052<br>0.098<br>0.040<br>0.029	[illegible]
Ranch Hand cohort; 989 servicemen responsible for aerial AO spraying in Vietnam	Henriksen et al., 1997 (6); Steenland et al., 2001 (1); and USAF, 2005 (7)	Incidence or prevalence of DM (depending on the analysis) defined as a verified post-deployment diagnosis or a 2-hr post-oral glucose challenge blood glucose of ≥200	Back-extrapolated levels for low and high exposure, current level for background*<br>Background (n=422); median =5.7<br>Low (n=284); median=52.7<br>High (n=283); median=197.5<br>* as reported in Henriksen et al., 1997 (6)<br><br>Back-extrapolated levels for all persons with serum TCDD ≥10 pg/g lipid*<br>10-64 (n=NR); midpoint=37<br>64-140 (n=NR); midpoint=102<br>140-376 (n=NR); midpoint=258	 <br>0.095<br>0.172<br>0.201<br><br><br><br><br>NR<br>NR<br>NR	[illegible]

Study; population description and total sample size	Reference(s)	DM measure and definition of diagnosis	TCDD exposure types, categories (N) and category specific measures of central tendency (pg/g lipid)	Proportion with DM (P_{DM})	M<br>(<br>a
		mg/dl	376-3290 (n=NR); midpoint=612†<br>* as reported in Steenland et al., 2001 (1)<br>† cutoff + range of the previous interval<br><br><u>Back-extrapolated (initial) levels *</u><br>Low 32-79 (n=140); midpoint=55.5<br>79-199 (n=142); midpoint=139<br>>199 (n=141); midpoint=319†<br>* as reported in USAF, 2005 (7)<br>† cutoff + range of the previous interval	NR<br><br><br><br><br><br>0.214<br>0.239<br>0.248	L<br>c<br>0<br>*<br>a<br>A<br>E<br>n<br><br>F<br><br><br>*<br>b<br>p<br><br>H<br>1<br>*<br>(<br>A<br>c<br>n
US Air Force cohort – comparison group for the Ranch Hand study; 1197 veterans who served in Vietnam but were not involved in Operation Ranch Hand	Longnecker et al., 2000 (8)	Incidence of DM defined as a verified post-service diagnosis or a 2-hr post-oral glucose challenge (100 gm) blood glucose of ≥200 mg/dl	<u>Current TCDD levels</u><br><2.8 (n=298); midpoint=1.4*<br>2.8-4.0 (n=305); midpoint=3.4<br>4.0-5.2 (n=295); midpoint=4.6<br>>5.2 (n=299); midpoint=6.4†<br>*Assuming lowest value is 0<br>† cutoff + range of the previous interval	 0.087<br>0.082<br>0.193<br>0.204	 C<br><br><br><br><br>L<br>c<br>0<br>A<br>v<br>n<br>s
Cross-sectional US Army Chemical Corps study; 662 Vietnam War veterans : who reported spraying herbicides	Kang et al., 2006 (9)	Prevalence of self-reported and medical record-confirmed DM	<u>Current TCDD levels (in 357 persons)</u><br>0.5-2.5 (n=178); midpoint=1.5<br>≥2.5 (n=179); midpoint=4.5*<br>*cutoff + range of the previous interval	 0.118<br>0.218	 P<br><br>*<br>b<br>p

Study; population description and total sample size	Reference(s)	DM measure and definition of diagnosis	TCDD exposure types, categories (N) and category specific measures of central tendency (pg/g lipid)	Proportion with DM (P_{DM})	M (a
Cross-sectional study of 1224 Korean Vietnam War veterans	Kim et al., 2003 (10)	Prevalence of DM, definition not provided	Current TCDD levels<br>Level I (n=307); mean=0.60<br>Level II (n=305); mean=0.62<br>Level III (n=306); mean=0.78<br>Level IV (n=306); mean=0.87	0.121<br>0.115<br>0.127<br>0.141	P<br>*<br>b<br>p
Cross-sectional study of the general population in Japan; 2,266 persons recruited from a variety of residential settings (cities, and farming and fishing villages) without occupational exposure to chlorinated compounds	Nakamoto et al., 2013 (11)	Prevalence of self-reported diabetes	Current TCDD levels<br><25th percentile (n=560), midpoint=0<br>25th-50th percentile (n=562), midpoint=0.5<br>50-75th percentile (n=565) midpoint=1.5<br>>75th percentile (n=558) midpoint=3.0*<br>* cutoff + range of the previous interval	0.004*<br>0.037*<br>0.060*<br>0.102*<br><br>* personal communication with study authors	C<br>A<br>s<br>h<br>y

Abbreviations: DM= diabetes mellitus, ICD = International Classification of Diseases; TCP = trichlorophenol, TCDD = tetrachlorodibenzodioxin; OR=odds ratio, PR = prevalence ratio, RR = risk ratio, HR=hazard ratio, BMI = body mass index, FHx = family history, WC = waist circumference

References

1. Steenland K, Calvert G, Ketchum N, et al. Dioxin and diabetes mellitus: an analysis of the combined NIOSH and Ranch Hand data. *Occup Environ Med* 2001;58(10):641-8.
2. Calvert GM, Sweeney MH, Deddens J, et al. Evaluation of diabetes mellitus, serum glucose, and thyroid function among United States workers exposed to 2,3,7,8-tetrachlorodibenzo-p-dioxin. *Occup Environ Med* 1999;56(4):270-6.
3. Zober A, Ott MG, Messerer P. Morbidity follow up study of BASF employees exposed to 2,3,7, 8-tetrachlorodibenzo-p-dioxin (TCDD) after a 1953 chemical reactor incident. *Occup Environ Med* 1994;51(7):479-86.
4. Ott MG, Messerer P, Zober A. Assessment of past occupational exposure to 2,3,7,8-tetrachlorodibenzo-p-dioxin using blood lipid analyses. *Int Arch Occup Environ Health* 1993;65(1):1-8.
5. Warner M, Mocarelli P, Brambilla P, et al. Diabetes, metabolic syndrome, and obesity in relation to serum dioxin concentrations: the Seveso women's health study. *Environ Health Perspect* 2013;121(8):906-11.
6. Henriksen GL, Ketchum NS, Michalek JE, et al. Serum dioxin and diabetes mellitus in veterans of Operation Ranch Hand. *Epidemiology* 1997;8(3):252-8.
7. USAF. Air Force Health Study Final Report: An Epidemiologic Investigation of Health Effects in Air Force Personnel Following Exposure to Herbicides. *Available: at http://wwwdticmil/cgi-bin/GetTRDoc?Location=U2&doc=GetTRDocpdf&AD=ADA438835 [accessed May 10, 2014]* 2005.

8. Longnecker MP, Michalek JE. Serum dioxin level in relation to diabetes mellitus among Air Force veterans with background levels of exposure. *Epidemiology* 2000;11(1):44-8.
9. Kang HK, Dalager NA, Needham LL, et al. Health status of Army Chemical Corps Vietnam veterans who sprayed defoliant in Vietnam. *Am J Ind Med* 2006;49(11):875-84.
10. Kim JS, Lim HS, Cho SI, et al. Impact of Agent Orange exposure among Korean Vietnam veterans. *Ind Health* 2003;41(3):149-57.
11. Nakamoto M, Arisawa K, Uemura H, et al. Association between blood levels of PCDDs/PCDFs/dioxin-like PCBs and history of allergic and other diseases in the Japanese population. *Int Arch Occup Environ Health* 2013;86(8):849-59.
